# Mr. Bellamy's Case of Fracture of the Cranium

**Published:** 1801-03

**Authors:** G. Bellamy

**Affiliations:** Surgeon. His Majesty's Ship Spencer, at Sea


### *To the Editors of the Medical and Phyfical Journal.*

Gentlemen,

In the following cafe of Fracture of the Cranium are fome unufual circumftances; and I think fome ufeful practical conclufions may be drawn therefrom. But it appears to me to militate againft our fuppofed theories of injuries of the head, and to prove how inadequate they are to explain the phyfiology and pathology of the brain. I am, &c.

*His Majefty's Ship Spencer, at Sea, Jan.* 3, 1800.

G. BELLAMY, Surgeon.

---

William Kirk, a feaman, aged about 40, on the 14th October, 1797, received by a fall from his hammock (his head ftriking againft the winch of the pump) a fracture of the external table, (it muft be remembered I fpeak fo far as the injury was difcovered at the time) and through the diploe of the left parietal bone.—Symptoms of debility, but none of compreffion.—I made a free incifion down to the pericranium on each fide of the wound, wound, (which was lacerated and much bruifed) to dilate it, and remove the tenfion and inflammation fo ufually attendant on partial divifion of the fcalp, particularly when inflicted by a blunt weapon; whereby I was alfo better able to trace the extent of the fracture. Though the accident happened on the 14th, at night, he did not complain till the 15th.—Simple dreffings were applied, and in the evening I gave him opii gr. ij. vin. lbfs. and lemonade, ad libit.—16th, Gave him vin. lbfs. ter de die; at night, oil antim. c. opio.—17th, No fymptoms of compreffion but of debility. Rep. ut antea.—18th, Vin. lbij. de die, and renewed the dreffings. The appearance of the wound very tolerable; no detached puffinefs of the fcalp or pericranum; but a fecond fmall wound near the other has alfo the bone bare.—19th, Repet.—20th, Ut antea; is doing well; is dreffed every other day.—21ft, Wine, &c.—22, Wine and opium; takes mutton broth and other food, having had no fever, and complaining of nothing but weaknefs.—23d, Ut antea.—24th, Continues his foup, wine and opium, occafionally; and his bowels have been pretty regular; have been once affifted by a cathartic, (in the form of pills) as he threw off a faline purge.—25th and 26th, R. U. A.—27th, The wound is healing faft, and particularly healthy; he has not had one ferious fymptom fince the injury; therefore hope no affection of the brain, efpecially as the fmaller wound is entirely healed; has had no vomitings, delirium or fever, alteration of the countenance or eye, nor pulfe otherwife than natural; complains only of a fhooting pain through the head, particularly at the forehead, with a fenfe of tremor paffing through the head, and towards the neck, and fome pain in the back, (likely from the blow when he fell); but all thefe fymptoms have leffened daily, and he has recovered fpirits and appetite.—28th, U. A. Was informed late this night of his having expreffed many incoherencies; a more than ufual defire of drink, and other marks of delirium. I immediately drew my opinion of what had taken place, and far without having expected it; dreading, though my hopes were fanguine, that infidious approach of fecondary inflammation of the brain, which often takes place weeks after the patient has every appearance of doing well. When I faw him in the morning I found his pulfe accelerated, and full; delirium; rather muttering than otherwife; complained of a fenfe of corded tightnefs round his head; his countenance and eyes rather turgid; in fhort, I concluded, without doubt, that all my hopes of primary recovery were vanifhed, and that now the brain was oppreffed; be it either from extravafation, (at the time of the accident, now beginning to irritate by corrofion the dura mater) or blood poured out fince, and acting as compreffion; or as inflammation communicated from from the ſcalp, and through the other coverings by the connection of parts to the membranes of the brain; but not from the leaſt poſſible idea of what was really the caſe, (which will be related); however, decidedly, the operation of the trepan was pointed out for the relief of the oppreſſed organ. For which purpoſe, on the 29th, in the morning, I took him on ſhore to Almada Hoſpital, near Liſbon. I would have wiſhed to perform the operation on board, but the numerous inconveniencies to which a patient with a dangerous wound is there expoſed need not be told; and after all (ſuffering from noiſe, and the want of many comforts) I muſt finally have removed him, when it could have been done with ſafety; but before which time he would have been endangered by various cauſes, which his immediate removal was intended to prevent. The fracture was about two inches long, lengthways of the bone, and towards the angle formed by the ſagittal and lambdoid ſutures. Mr. Grey, ſurgeon of the hoſpital, and myſelf, decided on what ſhould be done. Firſt, to make a free inciſion of the ſcalp, to examine the bone; (indeed, I could not convince him by the accurate obſervations I made at the time of the accident, that there was no evident depreſſion) that done, and examining the fracture, which admitted the head of the probe, and in one direction, paſſing it between the two tables poſteriorly, and rather obliquely inwards; opened a ſuſpicion of the internal table being depreſſed, (though this free paſſage of the probe might be from the caries of the bone, and from the expected exfoliation with which I had hopes all would have finiſhed well); but no ſymptom was excited by this probing of the wound; no ſenſe of vomiting or pain, however rudely preſſed:—However, there were ſuch ſymptoms, the only chance of relieving which was the uſe of the trephine; the immediate application of which I recommended to temporizing with, or to alleviating ſymptoms of inflammation by bleeding; particularly obſerving the time ſince the accident had happened, and debility gradually induced by laying in bed, a ſpare diet, &c. and as now, indeed, he was much more compoſed, and with every mark of oppreſſion; ſo that there were but two things to be done; either by ſuppoſing the delirium that had taken place to be accidental, in part the effect of the opium, and the impreſſion made on his mind laſt night by violent thunder and lightning, of which he was even whilſt in delirium mindful; and there are none of us but muſt know the ſympathy from the leaſt injury approaching the brain to external cauſes ſuch as thoſe; I ſay, by ſuppoſing theſe ſymptoms to be occaſional, and now diſappearing, either to go on with the treatment hitherto obſerved, and inſtead of evacuants to ſupport him by cordials under the debility with which he was oppreſſed; or elſe to trepan immediately, to remove whatever exciting caufe might be found. The latter was determined on, knowing the frequency of fuch deceitful cafes by which we are often unexpectedly deprived of a patient; and that the fymptoms were but too clearly marked of that fecondary affection of the brain, which fhows itfelf in the flow approach of difeafe. Mr. Grey did not think depreffion unlikely. I rather thought extravafation, or formation of matter;—indeed, I cannot conceive how a piece of bone fhall for a fortnight lay preffing on the brain, without caufing any one ferious fymptom, and then act (I fuppofe) merely as an irritant fubftance bringing on inflammation, &c.; for it could not be by depreffion only; elfe, why did not fymptoms of compreffion appear before? It would fhew us, that compreffion of itfelf was nothing, but only as it induced diforganization of the brain by inflammation as a foreign body, which it may do fooner or later according to the violence of the blow, the particular bearing of the oppreffive body, and predifpofing caufes. That fuch is the cafe, the following inftance will fhow; and the trueft conclufion I can make therefrom, is, that without waiting for fymptoms, compreffion of whatever kind muft be removed whenever the feat thereof is pointed out.

A crucial incifion was made, the pericranium removed, and a perforation made on the outer fide of the fracture, juft fo as to take in the circle of the fame. On removal the dura matter was found turgid, with blood veffels oppreffed; a grumous dark appearance like to a flight extravafation, but the whole of the circle defcribed by the trephine did not come away entire; that part of the upper table neareft the fracture remaining in a fplinter-like manner, rather loofe and depreffed. Now was feen the nature of the injury, which was a depreffion of the inner table, (independent of the outer) with a confiderable extent of fplintered detachment, more or lefs, (but not quite removed from the external table, and only by force of the bone nippers feparated one from the other) for the fpace of one inch in the direction of the fracture. This piece could not be removed without a fecond perforation, which was accordingly made on the outer fide of the fracture (the apparent direction of the fplintered piece). The circle being removed, and the two perforations being now as one, the whole detached piece was with *difficulty* removed, becaufe it had its ftricteft connection under thofe fides of the perforations which prefented the angles; that is, in the direction of the fracture, particularly anteriorly; whence a large fcale was with force withdrawn, as indeed was the whole, not only by the permanent fituation it feemed to have procured, (being very tight on the dura matter) but alfo from a particular inftance of, no doubt, increafed compreffion, by an addition of offeous offeous matter, which had been generated from the diploe of the depreffed piece, and grown up againft the outer table; by which refiftance was added, and at the fame time the deceit of feeling with the probe, which went feemingly no lower than the ufual depth of the deploe;—depreffion ftill exifting. Great caution was neceffary in removing the pieces, fearing to hurt the membranes. The bone had either carried down with it fome blood, (or it had there accumulated) a membranous-like attachment, which we feared, when forced by pulling away the bone, to be the dura matter. Had the fecond perforation been made in the direction of the fracture, the depreffed pieces would have been more eafily removed; and this fhould always be done, unlefs where otherwife pointed out. Being fatisfied fo far as prudence could determine of having removed all oppreffion, the flaps were laid down, and fuperficially dreffed only; not being decided on what might ftill be neceffary. An enema was adminiftered, the laxative ordered laft night not having operated. I confider this to be a fingular and interefting cafe. Nothing particular happened after the operation, and the man was foon perfectly cured.

