# Errata

**Published:** 1802-10-01

**Authors:** 


					ERRATA.
Vol. v. p. 25S, !. 36, for outer, read other.

				

